# Advances of new drugs bedaquiline and delamanid in the treatment of multi-drug resistant tuberculosis in children

**DOI:** 10.3389/fcimb.2023.1183597

**Published:** 2023-06-13

**Authors:** Hanzhao Zhu, Xintong Zhou, Zengfang Zhuang, Lianju Li, Jing Bi, Kaixia Mi

**Affiliations:** ^1^ Chinese Academy of Science (CAS) Key Laboratory of Pathogen Microbiology and Immunology, Institute of Microbiology, Chinese Academy of Sciences, Beijing, China; ^2^ Savaid Medical School, University of Chinese Academy of Sciences, Beijing, China; ^3^ School of Basic Medicine, Shandong First Medical University and Shandong Academy of Medical Sciences, Jinan, China; ^4^ Baoding Hospital of Beijing Children’s Hospital, Capital Medical University, Baoding Key Laboratory for Precision Diagnosis and Treatment of Infectious Diseases in Children, Baoding, China

**Keywords:** tuberculosis (TB), multi-drug resistant TB, children, bedaquiline, delamanid

## Abstract

Tuberculosis (TB) is a major public health problem, with nearly 10 million new cases and millions of deaths each year. Around 10% of these cases are in children, but only a fraction receive proper diagnosis and treatment. The spread of drug-resistant (DR) strain of TB has made it difficult to control, with only 60% of patients responding to treatment. Multi-drug resistant TB (MDR-TB) is often undiagnosed in children due to lack of awareness or under-diagnosis, and the target for children’s DR-TB treatment has only been met in 15% of goals. New medications such as bedaquiline and delamanid have been approved for treating DR-TB. However, due to age and weight differences, adults and children require different dosages. The availability of child-friendly formulations is limited by a lack of clinical data in children. This paper reviews the development history of these drugs, their mechanism of action, efficacy, safety potential problems and current use in treating DR-TB in children.

## 1 Introduction

Tuberculosis (TB) is a major public health problem that is becoming increasingly concerning in children, with 1.1 million new cases accounting for 11% of all new TB cases worldwide in 2021 (World Health Organization, 2022a). Multi-drug resistant/rifampicin-resistant (MDR/RR) TB is one of the leading causes of death worldwide and has been drawn attention for its severe form in children. The diagnosis and treatment of TB in children are particularly difficult due to their developmental stage, so it is important to understand the opportunities and challenges currently facing TB in children, as well as MDR/RR-TB infection in particular. To reduce the burden of this disease on children’s health, improved early diagnosis methods and precise and effective treatments must be developed if we are to achieve our goal of “zero death from tuberculosis in children” (World Health Organization et al., 2013).

Drug-resistant tuberculosis (DR-TB) is a growing problem that makes it more difficult to achieve disease control goals. MDR-TB is a type of TB that is resistant to two important drugs, rifampicin and isoniazid (World Health Organization, 2022b). In 2021 alone, there were 450,000 new patients with MDR-TB (World Health Organization, 2022a). An estimated 25,000 to 32,000 children develop MDR/RR-TB each year (Jenkins et al., 2014; Dodd et al., 2016; Jenkins and Yuen, 2018). MDR/RR-TB treatment cases are 2.5% of the total number of children with MDR/RR-TB starting treatment and only 10.1-12.9% of the estimated number of children with MDR/RR-TB (World Health Organization, 2020). MDR/RR-TB is difficult to treat due to the lack of effective drugs, which are costly and require a long treatment period (Dodd et al., 2016; Guglielmetti et al., 2021).

**Figure 1:**
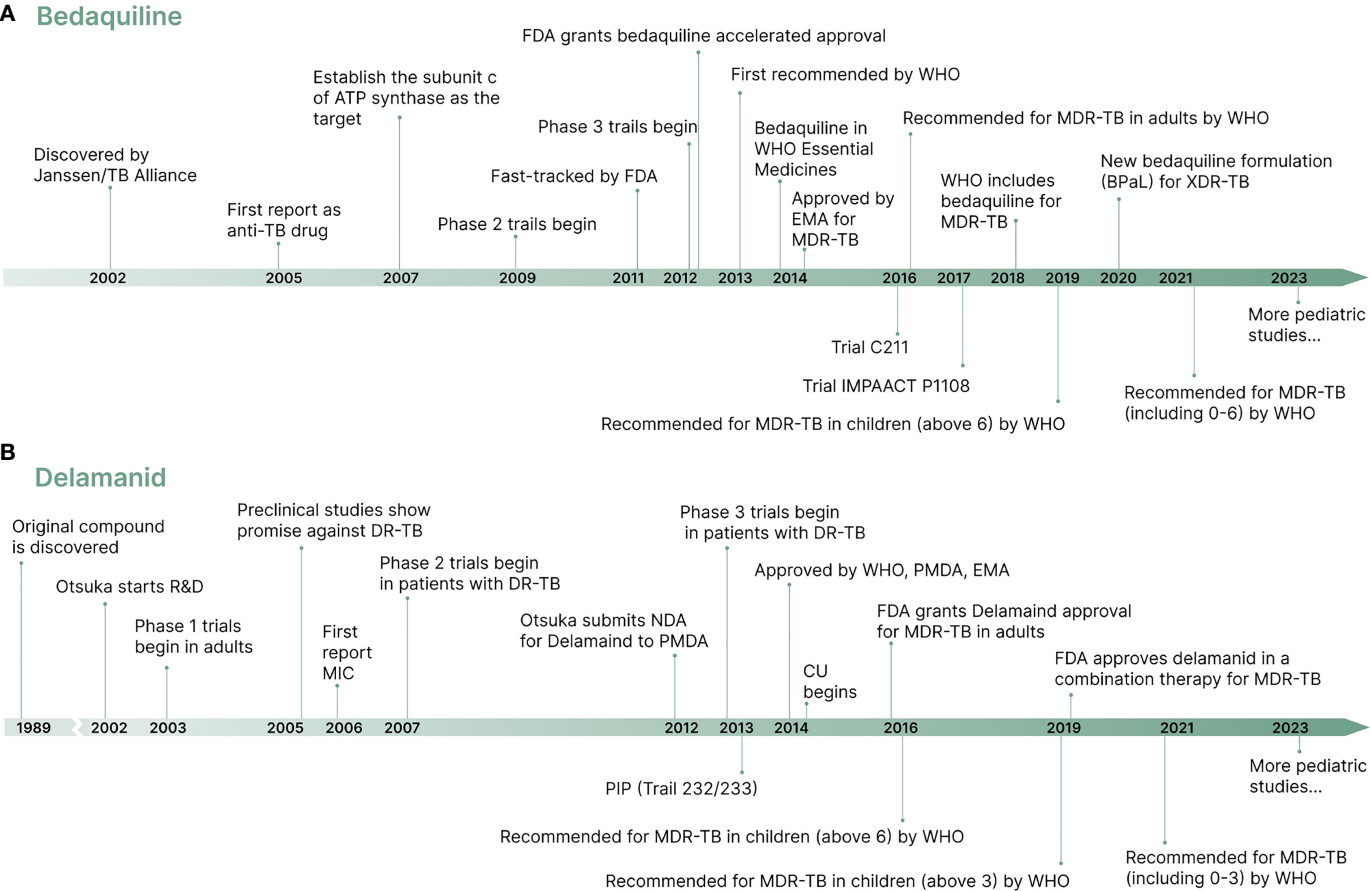
A brief timeline of the development of new drugs bedaquiline and delamanid. **(A)** Bedaquiline development timeline. **(B)** Delamanid development timeline.

Treatment of MDR-TB is challenging due to a limited number of effective drugs, associated with high drug burden cost, long treatment duration, and potential for adverse effects (Chan et al., 2013). Studies showed that MDR-TB treatment drugs such as aminoglycosides can cause irreversible ototoxicity, hepatotoxicity, and neurological side effects (Dheda et al., 2017; Liu et al., 2018). After fifty years of stagnation in identifying new targets and drugs to treat TB (Zumla et al., 2013), new drugs, bedaquiline and delamanid, have showed promising efficacy in adults (Skripconoka et al., 2013; Borisov et al., 2017; Guglielmetti et al., 2017a; Mohr et al., 2018). Significant advances have been made in treating MDR-TB in children after years of neglect in developing and using of these new drugs (Sandgren et al., 2012). Shown in **Figure 1** is the development history of bedaquiline and delamanid used in the treatment of MDR-TB. The timeline provides an overview of the drug development process, from discovery to regulatory approvals, including preclinical studies, clinical trials for both adults and children. This information highlights the significant efforts put into bringing these drugs to the market, and their potential to revolutionize the treatment of MDR-TB.

In 2022, the World Health Organization (WHO) revised the guidelines for TB treatment in children, recommending the use of new drugs bedaquiline and delamanid to treat MDR-TB (World Health Organization, 2022b). In this review, we will provide an overview of the progress made in treating children with MDR-TB using bedaquiline and delamanid, including their efficacy, safety, and treatment recommendations.

## 2 Treatment of TB in children using of the new drug bedaquiline and delamanid

Historically, the treatment of TB in children has lagged behind that of adults, largely due to a lack of research specifically tailored to young people. For instance, the duration of TB treatment in children was based on the adult studies, mandating a 6-month combination of daily medications. However, a recent study has recommended that the treatment duration for children with drug-susceptible (DS) TB be shortened from 6 months to 4 months (Turkova et al., 2022). In line with this, the WHO has updated the recommendations for the management of TB, including MDR/RR-TB in children. These recommendations include the use of bedaquiline in shorter or longer regimens for treating MDR/RR-TB in children of all ages, and the use of delamanid in longer regimens for treating MDR/RR-TB in children of all ages (World Health Organization, 2022b). The WHO provides guidelines for the dosage recommendations for drugs used in MDR-TB regiments, which are based on body weight and age. Details can be found on the WHO website and are described in full at https://www.ncbi.nlm.nih.gov/books/NBK539514/table/annex2.tab2/. It is important to select the most effective and appropriate treatment plan for each age group. Children are generally expected to respond to TB treatment as well as or better than adults (Ettehad et al., 2012; Donald et al., 2013; Nachman et al., 2015). However, there is a lack of research specifically targeting children, and there are only a limited number of drug regimens available for children. Further research is needed on the effectiveness of different treatment regimens in this group of children.

While the basic principles for designing treatment regimens for children with TB are largely similar to those used for adults (Schaaf and Marais, 2011), there are some differences in the pharmacokinetics and toxicity of drugs when they are administered to children. This makes it challenging to determine which treatments are most effective and appropriate for children, as it is not always possible to directly translate drug dosing recommendations from adults to children.

Further research is needed to determine the efficacy of different children-specific treatment plans to ensure optimal care for children with TB (Poorana Ganga Devi and Swaminathan, 2013). Despite the challenges in the provision of children TB formulations, new drugs like bedaquiline and delamanid have been developed for children with TB. Innovative approaches like these can accelerate the development of more effective treatments tailored to children.

## 3 Bedaquiline

Bedaquiline, developed by Johnson & Johnson’s Janssen Pharmaceuticals, was approved for use in adults with MDR-TB in 2012 (Mahajan, 2013). Its discovery dates back to 2005, when Andries et al. investigated the inhibitory effect of various chemicals on the growth of *M. smegmatis*. They found that bedaquiline, then known as TMC207, had a potent inhibitory effect on several species of mycobacteria, particularly *M. tuberculosis*, due to its ability to inhibit ATP synthase, which is essential for energy production in *M. tuberculosis* (Andries et al., 2005). Blocking the enzyme responsible for ATP synthesis, bedaquiline has demonstrated a reduction in the growth and survival of MDR-TB strains, making it an effective treatment option for TB in children (Andries et al., 2014; Diacon et al., 2014). Bedaquiline is the first new drug approved for the treatment of MDR-TB since rifampin in 1971 (Mahajan, 2013). In the WHO Consolidated Tuberculosis Guidelines (2022), bedaquiline has been conditionally recommended for treating children with MDR-TB aged less than 6 years (World Health Organization, 2022b). Although bedaquiline has many advantages as a promising new adult TB drug, more research is required to understand how young children are affected by bedaquiline and what dose to use for them.

### 3.1 Mechanisms and pharmacokinetics of bedaquiline

Bedaquiline is a new anti-TB drug that functions by blocking the ion-binding sites of the mycobacterial ATP synthase, which converts ADP to ATP *via* a transmembrane electrochemical ion (H^+^ or Na^+)^ gradient, thereby producing energy. Bedaquiline specifically targets the ion-binding sites present in the c-subunit of ATP synthase, which hinders the proton pump and results in a decline in intracellular ATP levels (Andries et al., 2005; Koul et al., 2007). Additionally, bedaquiline also targets the ϵ-subunit of F-ATP synthase by interacting with Trp16 residues (Kundu et al., 2016). By inhibiting mycobacterial ATP synthase, bedaquiline leads to ATP depletion, thereby damaging energy-producing mycobacteria and disrupting pH homeostasis, ultimately inhibiting strain growth. Unlike other quinolones antibiotics that block DNA gyrase, bedaquiline specifically targets the mycobacterial ATP synthase (Andries et al., 2005). Additionally, bedaquiline has a high degree of specificity to mycobacterial ATP synthase compared to other organisms, such as humans or mice. Because bedaquiline has low sensitivity to mitochondrial ATP synthases of humans, it is unlikely to cause toxicity (Haagsma et al., 2009). Studies have shown that bedaquiline exhibits superior *in vitro* antibacterial activity when compared to other antibiotics. This highlights ATP synthase as a promising target for the treatment of TB, as it offers a new mode of action against TB. This differs from traditional antibiotics such as quinolones, which block DNA gyrase (Andries et al., 2005; Diacon et al., 2009; van Heeswijk et al., 2014). Furthermore, bedaquiline does not cross-reaction with other anti-tuberculosis drugs, making it effective against both MDR-TB strains and DS-TB strains (Andries et al., 2005).

Human pharmacokinetic studies have shown that bedaquiline is well absorbed by the body when taken orally with food (Rouan et al., 2012; Diacon et al., 2013; D’Ambrosio et al., 2017). A standard diet containing approximately 22 g of fat has been found to improve bedaquiline bioavailability by a factor of 2 compared to fasting U.S. Food and Drug Administration, (2012). This oral administration method has been used in the treatment of TB. Bedaquiline has a linear relationship between dose and maximum plasma concentration (C_max_), and peak concentration is reached on average 5-6 hours after treatment, and an effective half-life of 24 hours on average (Andries et al., 2005; Rouan et al., 2012; Diacon et al., 2013; D’Ambrosio et al., 2017). Drug efficacy is related to cumulative weekly doses, suggesting intermittent administration is favorable.

Bedaquiline is mainly metabolized by cytochrome P-450 3A4 (CYP3A4) and is converted to the relatively inactive N-desmethyl metabolite N-monodesmethyl (M2) (Dooley et al., 2012). *In vitro* studies have shown that M2 can cause more disruption of cellular phospholipid deposition than bedaquiline, which may result in adverse effects such as QT interval prolongation and hepatotoxicity (van Heeswijk et al., 2014). However, *in vivo* studies with M2 and bedaquiline have not shown any such side effects, even at C_max_ (Dooley et al., 2012).

Drug resistance is a growing concern in the use of antibiotics, including bedaquiline. The identification of bedaquiline-resistant strains in MDR-TB patients without prior exposure to bedaquiline (Villellas et al., 2017; Xu et al., 2017) suggests that resistance to this drug can emerge spontaneously. This highlights the importance of careful monitoring of patients receiving bedaquiline and the need for further research to understand the mechanisms of resistance and to develop strategies to prevent or overcome it. The emergence of bedaquiline-resistant strains underscores the need for continued research and development of new treatment options for MDR-TB in both adult and children.

### 3.2 Bedaquiline recommended for the treatment of MDR-TB

A clinical study was conducted to evaluate the efficacy of bedaquiline, when added to the standard treatment for DR-TB. The study found that the addition of bedaquiline significantly reduced the amount of time for patients’ sputum to convert to culture when compared to a placebo. When the patients were followed up for an additional 24 weeks, 50% of the patients in the bedaquiline group achieved culture conversion in 78 days, whereas 129 days in the placebo group (Diacon et al., 2009; Diacon et al., 2012). Similarly, another clinical trial involving patients with DR-TB showed that patients who received bedaquiline had higher rates of sputum conversion compared to those who received a placebo. Specifically, after 24 weeks, 79% of patients in the bedaquiline group achieved sputum conversion compared to 58% in the placebo group (Lakshmanan and Xavier, 2013). In addition, the TMC207-C208 Clinical Trials (NCT00449644) evaluated sputum-culture conversion in patients and found that adding bedaquiline to standard TB treatment for 24 weeks resulted in faster recovery than placebo. After 120 weeks, patients who received bedaquiline showed significantly more improvement compared to those who received a placebo (Diacon et al., 2014). A study of 20 patients with MDR/extensively-drug resistant (XDR)-TB showed that 12 patients (60%) achieved culture conversion within 8 weeks and 20 patients (100%) achieved culture conversion within 6 months when treated with a bedaquiline regimen for at least 6 months (Olaru et al., 2017). A larger group study that looked at treatment outcomes of 428 patients with DR-TB in 15 countries found that 62.4% were cured at the end of their treatment, 13.4% died during treatment, 7.3% dropped out of treatment before completion, and 7.7% did not successfully respond to the prescribed regimen (Borisov et al., 2017). Several studies have supported a significant reduction in the mortality rate of patients who received bedaquiline compared to those who received a placebo (Ahmad et al., 2018; Olayanju et al., 2018; Schnippel et al., 2018; Mbuagbaw et al., 2019). The available evidence suggests that bedaquiline is effective in treating DR-TB. It has been classified by the WHO as the drug of choice for this disease and is recommended for use in all-oral treatment regimens (World Health Organization, 2022b).

### 3.3 Side effects of bedaquiline

The safety of bedaquiline has been assessed and it is generally considered safe for long-term use with mild side effects such as nausea and headache (Guglielmetti et al., 2017b). However, it can cause QTc prolongation (Darmayani et al., 2022) and caution should be used when combining with other drugs that have similar risks.

In an early evaluation of bedaquiline’s adverse effects in 75 patients, Rustomjee et al. found that the administration of 400 mg bedaquiline daily led to very few mild adverse events, including hemoptysis (21.4%), rash (7%), diarrhea (7%), and somnolence (7%) (Rustomjee et al., 2008). Adverse events, mainly related to other drugs in the regimen, occurred in 23 of 75 patients (77%) in the study by Olaru et al. Although QT interval prolongation was observed in 95% of patients, no arrhythmias or deaths were recorded, and none had to discontinue treatment due to bedaquiline-related adverse events (Olaru et al., 2017). A case analysis of 247 MDR-TB patients conducted by Borisov et al. showed that 5.8% discontinued bedaquiline treatment due to adverse events and there was one death, not considered related to bedaquiline use (Borisov et al., 2017). A retrospective analysis by Hewison et al. showed that 84.4% achieved a culture conversion rate, 79.3% experienced some form of the adverse event, and 10 died, two of which may have been related to bedaquiline use (Hewison et al., 2018).

Overall, studies have shown that bedaquiline has a low incidence of adverse events and a high rate of culture conversion, indicating bedaquiline is generally safe and well-tolerable with mild side effects. In the long term, bedaquiline has a potential for adverse effects, including the possibility of death. Consequently, caution should be taken when combining bedaquiline with other drugs that carry similar risks, such as fluoroquinolones or CYP3A4 inhibitors. QTc prolongation may occur. In contrast, levofloxacin, a fluoroquinolone drug, has been found to have fewer drug interactions and cause less QTc prolongation (Harausz et al., 2017; Denti et al., 2018). As such, long-term follow-up of patients is necessary to monitor for any life-threatening adverse effects.

### 3.4 Safety and effectiveness of bedaquiline in children with drug-resistant TB

The use of bedaquiline in the treatment of DR-TB in children has been limited due to its potential for adverse side effects. While clinical trials are ongoing to assess its safety and efficacy, information on its use in children is scarce because they are often not included in trials. A compassionate use study of bedaquiline to treat MDR-TB in children under 15 indicated that bedaquiline was associated with higher success rates in eliminating TB infection after 24 weeks of treatment. This supports the use of bedaquiline as a treatment option and suggests that bedaquiline is an effective regimen for treating TB in children (Guglielmetti et al., 2015). Studies have also assessed the potential mortality associated with the use of bedaquiline. An analysis of TB cases registered in EDRweb, excluding children under 15, reported that among 1016 MDR-TB patients treated with bedaquiline, there were 128 deaths (12.6%), a lower mortality rate than standard treatment (24.8%) (Schnippel et al., 2018). The study by Koirala et al. found that 74.2% of patients had a successful treatment outcome with the use of bedaquiline, with only 6.5% dying and 2.9% experiencing a failure rate due to its use (Koirala et al., 2021). Another study looked at 27 young patients with MDR-TB and found that although 5 of them experienced QTc prolongation, it was still safe to continue treatment with bedaquiline alongside drugs that can cause heart problems (Achar et al., 2017). In addition, the safety and efficacy of bedaquiline were evaluated in 15 children, 3 of whom were HIV infected. Of these children, 10 experienced mild QTc prolongation and 2 experienced arthritis. The study also found that the bioavailability of bedaquiline in children was about 57% compared to what it was in adults, which could be partially explained by delaying feeding after administering bedaquiline on days when blood samples were taken for evaluation (Hughes et al., 2022).

Two clinical trials, Janssen C211 and IMPAACT P1108, have been investigating the treatment of pediatric patients with DR-TB since 2016 and 2017 respectively. These trials focus on pediatric MDR-TB patients in different age groups (Moodliar et al., 2021; https://www.impaactnetwork.org/studies/p1108). Despite limited data and small sample sizes, bedaquiline was found to be safe for heart health in both adults and children.

The WHO recommends that children under 6 years of age with TB receive all-oral treatment, with doses adjusted for age and weight (World Health Organization, 2022b). The treatment success rates of children who receive an all-oral regimen are comparable to those who do not receive it. Bedaquiline, having demonstrated efficacy and safety in younger age groups, may be used as an alternative to injectable drugs (Seddon et al., 2018).

Janssen is developing a child-friendly version of bedaquiline, but it may not be available for some time (Garcia-Prats et al., 2018). The practical dose of bedaquiline for children may be supported by the suspension or syrup forms of bedaquiline that are proven to be available (Taneja et al., 2023). Meanwhile, a trial (NCT03032367) sponsored by the IMPAACT Network is comparing the effectiveness of using whole tablets and dissolved bedaquiline for children who cannot swallow whole tablets (Garcia-Prats et al., 2018). Another study of 24 healthy adult volunteers found that bedaquiline tablets dissolved in water worked as well as those swallowed whole (Svensson et al., 2018). According to the WHO, until a child-friendly version is available, the current formulation can still be used to treat MDR-TB in children.

## 4 Delamanid

Delamanid (OPC-67683), a bicyclic nitroimidazole, is a new TB drug developed by Otsuka that has demonstrated potent TB activity *in vitro* and *in vivo* (Matsumoto et al., 2006; Saliu et al., 2007; Igarashi, 2017). It was originally discovered in 1989, and improvements have been made to eliminate mutagenicity while increasing its anti-TB effects (Ashtekar et al., 1993; Sasaki et al., 2006). Delamanid has the potential to be used as a treatment for TB because it does not show cross-resistance or antagonistic activities with other existing drugs such as isoniazid and rifampicin (Lewis and Sloan, 2015). It has been recommended by the WHO for use in longer regimens for the treatment of MDR-TB in children.

### 4.1 Mechanisms and pharmacokinetics of delamanid

Delamanid is an antibiotic drug used to treat MDR/RR-TB. Delamanid is a prodrug that works by reducing the nitro group of the F420-dependent nitroreductase, which activates the drug. This process also generates many intermediates that facilitate its action against MDR/RR-TB bacteria, making it more effective in treating mycobacterial infections (Field, 2013; Liu et al., 2018). Delamanid inhibits the production of mycolic acid, which is necessary for *Mycobacterium avium* survival and cell wall formation. By limiting the amount of mycolic acid produced, *M. avium* reproduction is inhibited, and the abnormal cell wall allows the drug to penetrate the cell. This makes delamanid more effective in treating mycobacterial infection because mycolic acids are only found in the cell walls of mycobacteria (Glickman and Jacobs, 2001; Matsumoto et al., 2006; Field et al., 2012; Ryan and Lo, 2014). Delamanid has inhibitory effects on *M. avium*, which may involve the release of free radicals such as nitric oxide (NO). These free radicals are essential in mammalian defense mechanisms against mycobacterial infections, making delamanid more effective in treating such infections (MacMicking et al., 1997; Xavier and Lakshmanan, 2014).

Tanneau et al. established a population pharmacokinetic (PK) model, investigated potential pharmacological interactions with bedaquiline, and determined the concentration-time course of delamanid and DM-6705 in adults with DR-TB. Plasma albumin concentration had no discernible effect on delamanid metabolism. Delamanid PK was not affected by the co-administration of bedaquiline (Tanneau et al., 2022). Using a large body of delamanid clinical data, a PK model for delamanid was developed in individuals with pulmonary MDR-TB. The model had a good fit and sufficient predictive power, taking into account different dosing populations and treatment regimens. Based on this model, the current recommended dose of 100 mg is more acceptable after analyzing the bioavailability of 100 mg, 200 mg, 250 mg, and 300 mg. However, given the number of drugs used to treat MDR-TB, it is critical to adjust the optimized dose in complex situations. The study was conducted in patients aged 18-64 years with MDR-TB, and pediatric data are still lacking (Wang et al., 2020).

A population pharmacokinetic analysis of delamanid and its major metabolite DM-6705 gained insight into the pharmacokinetic profile of these drugs in children with MDR-TB. This analysis utilized data from two clinical trials, involving participants aged 0.67 to 17 years, to provide healthcare providers with a better understanding of how children absorb and metabolize delamanid. The findings of this study can aid healthcare providers in adjusting dosages appropriately to achieve optimal treatment outcomes (Sasaki et al., 2022). Two clinical trials (NCT01856634 and NCT01859923) were conducted to evaluate the pharmacokinetics, safety, efficacy, and appropriate dosing of delamanid in MDR-TB children aged 0-17. The study included a total of 37 children who were grouped into four categories: 12-17, 6-11, 3-5, 0-2. These children received doses of 100 mg, 50 mg, 25 mg, and 5-10 mg respectively, twice daily. A favorable response was observed in 33 out of 37 children (89.2%) at 24 months. The safety profile of delamanid was found to be similar in children aged 0-17 years and adults who took delamanid (Garcia-Prats et al., 2018). The IMPAACT, 2005 trial is currently underway to evaluate the pharmacokinetics, safety, and tolerability of delamanid in combination with an optimized multidrug background regimen (OBR) for treating of MDR-TB in HIV-infected and uninfected children. The trial is expected to be completed by 2027, and can track its progress on the IMMPACCT Network website (https://test.impaactnetwork.org/studies/impaact2005). Moreover, in a lead-in pediatric PK study, the PHOENIx trial (NCT03568383), is evaluating once-daily delamanid dosing in children. More details about the trial can be found on the ClinicalTrials.gov website (https://clinicaltrials.gov/ct2/show/NCT03568383).

Delamanid has low water solubility, which can impede its absorption into the body. Nevertheless, animal studies have shown that its oral bioavailability ranges from 35 to 60% and it increases with food intake, particularly high-fat foods (Lewis and Sloan, 2015). Furthermore, delamanid has been found to have no interactions with CYP enzymes (Liu et al., 2018) and a low potential to interact with antiretroviral drugs. Consequently, it is feasible to co-administer delamanid with antiretroviral drugs without worrying about drug interactions (Mallikaarjun et al., 2016).

Besides its limited bioavailability, mutations in the *ddn*, *fgd1*, *fbiA*, *fbiB*, *fbiC* genes linked to the F420-dependent bioactivation pathway can result in delamanid resistance (Nguyen et al., 2020; Liu et al., 2022). This implies that some bacteria may develop resistance to delamanid therapy, necessitating the use of alternative drugs. Healthcare providers should monitor for any indications of resistance while prescribing this medication to ensure effective treatment outcomes.

### 4.2 Delamanid recommended for the treatment of MDR-TB

In 2014, Otsuka launched the first compassionate use (CU) program, which aimed to provide free-of-cost delamanid to patients with limited treatment options. This program also allowed the combination of delamanid and bedaquiline under certain circumstances. Studies have shown that 79% of patients treated with delamanid achieved culture conversion, which is a better outcome than those not treated with the combination of bedaquiline and delamanid (Hafkin et al., 2019).

In a phase II study, Gler et al. investigated the efficacy of delamanid for the treatment of pulmonary MDR-TB in 481 patients. The treatment regimen was supplemented with 100 mg or 200 mg of delamanid or placebo twice daily for 8 weeks. The results showed that the experimental group receiving delamanid had a higher rate of sputum transformation than the control group, indicating the potential as an effective therapy for MDR-TB (Gler et al., 2012). According to Skripconoka et al., a clinical trial study was conducted on 421 patients who were administered delamanid at dosages of 100 and 200 mg twice daily. The results reported that the patients who received delamanid for 6 months experienced a mortality rate of 1%, while those who received the drug for 2 months experienced a higher mortality rate of 8.3% (Skripconoka et al., 2013). A study from South Korea reported that 32 patients with MDR-TB received delamanid for 24 weeks. The study showed that 72.2% of the patients achieved solid media conversion and 50% achieved liquid media conversion after 8 weeks of treatment. After 24 weeks of delamanid treatment, the culture conversion rates significantly improved, with 94.4% and 92.9%, respectively. No serious adverse events or deaths were reported during the treatment period, indicating that delamanid can be safely used for long-term treatment of MDR-TB infection and is effective in achieving the culture conversion rates associated with successful treatment outcomes (Mok et al., 2018). A longer treatment regimen with delamanid may be more effective in reducing mortality associated with MDR-TB infection.

The studies concluded that delamanid is an effective anti-TB drug that can improve the culture conversion rates leading to successful therapeutic outcomes. The studies also indicate that delamanid is safe for long-term use. Delamanid should be considered as a potential treatment option for patients with MDR-TB who are unresponsive to conventional drugs or have limited alternatives.

### 4.3 Side effects of delamanid

Delamanid can cause QTc prolongation, anorexia, gastritis, malaise, anemia, and psychiatric disorders. In children, it may result in liver damage and low white blood cell counts. Monitoring for these side effects is necessary to ensure the safe use of delamanid (Skripconoka et al., 2013; Mohr et al., 2018). Although delamanid may cause increased QTc prolongation, it may not necessarily be associated with any serious health risks. Vomiting, QTc prolongation, and myalgia were the most commonly reported side effects of delamanid administration. In more severe cases, QTc prolongation could indicate serious health risks (Gler et al., 2012; Skripconoka et al., 2013; Mohr et al., 2018). For example, Hughes et al. conducted a study to determine the adverse effects of the delamanid regimen in 58 RR-TB patients in South Africa. The study found that vomiting, QTc prolongation, and myalgia were the most common adverse reactions reported with delamanid administration. Moreover, one patient exhibited progressively severe QTc prolongation and cardiac symptoms such as chest discomfort, lightheadedness, and palpitations (Hughes et al., 2019), which could indicate more serious health risks from taking the drug. Since delamanid is considered to be an effective drug, it should not be stopped abruptly stopped or discontinued due to QTc prolongation. Monitoring for side effects while taking delamanid is important to ensure its safe use.

One of the major concerns associated with the prolonged utilization of delamanid is its low water solubility. This problem results in suboptimal absorption of delamanid formulations in clinical trials (Tao et al., 2019). Limited bioavailability leads to more frequent dosing, which can increase the overall treatment expenses and affect the efficacy of the drug. Attempts have been made to optimize the delamanid formulation to increase its bioavailability and reduce dosing frequency. To improve the effectiveness of delamanid, it may be necessary to increase food intake or employ other measures such as liposomal formulations or co-administration with other drugs to ensure its effective delivery and absorption into the body. In a study aimed at optimizing the route of administration, an indigestible nanostructured lipid formulation was found to absorb duration of delamanid, outperforming milk or suspension formulations (Ramirez et al., 2021). This finding could be beneficial in reducing treatment costs for patients in low-income regions by reducing the number of doses required for successful outcomes. Additionally, a recent study has shown the *in vitro* efficacy of delamanid can be improved, and its water solubility increased, through cyclodextrin complexation (Patil et al., 2023).

### 4.4 Safety and effectiveness of delamanid in children with Drug-Resistant TB

According to the WHO’s comprehensive Tuberculosis Guidelines evaluate, delamanid is evaluated as part of a long-term regimen for the treatment of MDR/RR-TB in children. In a study, cultures were found to be negative in 116 (79%) out of 147 MDR-TB patients after 24 weeks of delamanid treatment. This included a negative culture rate of 20 of 25 (80%) in pediatric culture. 8% of participants experienced QTc prolongation, but no other serious adverse events were reported. These results suggest that delamanid can be an effective and safe therapy for the treatment of MDR-TB infection in both adults and children (Ghosh et al., 2021). The pharmacokinetics and safety of delamanid when administered to children with MDR-TB aged 0-17 years were evaluated in two clinical trials (NCT01856634 and NCT01859923) to determine the appropriate dose for this age group. The study results indicated that a good therapeutic response was achieved by 89.2% of patients at 24 months after the first dose. Furthermore, the safety profile of delamanid was found to be similar between adults and patients aged 0-17 years (Garcia-Prats et al., 2022).

According to the study conducted on adult volunteers, it has been shown that the bioavailability of dispersed 50 mg delamanid tablets is equivalent to that of whole tablets. This could be an alternative for patients who are unable to swallow whole tablets, such as children and other patients (Zou et al., 2023).

Despite its potential side effects, delamanid may still be considered a priority drug for certain demographic groups and for patients refractory to standard medications.

## 5 Combination the new drugs bedaquiline and/or delamanid with other drugs

Recent studies have shown encouraging results in the treatment of multidrug-resistant tuberculosis (MDR-TB) through the combined use of bedaquiline and delamanid. Extensive trials combining these drugs with other promising medications have provided data that can guide the treatment of MDR-TB in children (**Table 1**).

**Table 1:** The BDQ and/or DLM combination dosing clinical trials for TB treatment on ClinicalTrials.gov.

ClinicalTrials.gov Identifier	Conditions or disease	Age	Enrollment	Intervention/treatment	Phase	Trial Status	Start Date	Completion Date	the source of the clinical trial details information
NCT01215851	Pulmonary TB	18-65	85	PMD, PZA, BDQ, EMB, MXF	II	Completed	Oct 2010	Aug 2011	https://clinicaltrials.gov/ct2/show/NCT01215851
NCT01341184	TB	18-45	33	RFB, RFP, BDQ	I	Completed	Oct 2011	May 2012	https://clinicaltrials.gov/ct2/show/NCT01341184
NCT01691534	Pulmonary TB	18-65	105	PMD, BDQ, PZA, CFZ, EMB	II	Completed	Oct 2012	May 2013	https://clinicaltrials.gov/ct2/show/NCT01691534
NCT02193776	TB	18-75	240	BDQ, PMD, MXF, PZA, INH, EMB, RFP	II	Completed	Oct 2014	Feb 2018	https://clinicaltrials.gov/ct2/show/NCT02193776
NCT02216331	TB	19-55	32	RFT, RFP, BDQ	I	Completed	Mar 2010	May 2010	https://clinicaltrials.gov/ct2/show/NCT02216331
**NCT02333799**	**Pulmonary TB, MDR-TB, XDR-TB**	**≥14**	**109**	**PMD, BDQ, LNZ**	**III**	**Completed**	**Feb 2015**	**Aug 2020**	https://clinicaltrials.gov/ct2/show/NCT02333799
**NCT02409290**	**MDR-TB**	**≥15**	**588**	**MXF, CFZ, EMB, PZA, INH, PTH, KAN, LVX, BDQ**	**III**	**Active, not recruiting**	**Apr 2016**	**Estimated Apr 2023**	https://clinicaltrials.gov/ct2/show/NCT02409290
NCT02454205	TB, MDR-TB, XDR-TB	≥18	154	LNZ, BDQ, LVX, PZA, INH, ETA, TRD, MXF, KAN	II/III	Completed	Nov 2015	Aug 2021	https://clinicaltrials.gov/ct2/show/NCT02454205
NCT02583048	TB, HIV Infections	≥18	84	BDQ, DLM, DTG	II	Completed	Oct 2016	Feb 2021	https://clinicaltrials.gov/ct2/show/NCT02583048
**NCT02589782**	**Pulmonary TB, MDR-TB, XDR-TB**	**≥15**	**552**	**BDQ, PMD, MXF, LNZ, CFZ**	**II/III**	**Active, not recruiting**	**Jan 2017**	**Estimated Dec 2022**	https://clinicaltrials.gov/ct2/show/NCT02589782
NCT02619994	TB, MDR-TB	19-85	238	LNZ, DLM, LVX, PZA	II	Recruiting	Jan 2016	Estimated Jun 2021	https://clinicaltrials.gov/ct2/show/NCT02619994
**NCT02754765**	**Pulmonary TB, MDR-TB**	**≥15**	**754**	**BDQ, CFZ, MXF, LVX, PZA, LNZ, DLM**	**III**	**Active, not recruiting**	**Dec 2016**	**Estimated Sep 2023**	https://clinicaltrials.gov/ct2/show/NCT02754765
**NCT03086486**	**Pulmonary TB, MDR-TB, XDR-TB, Pre-XDR-TB**	**≥14**	**180**	**BDQ, PMD, LNZ**	**III**	**Active, not recruiting**	**Nov 2017**	**Estimated Feb 2022**	https://clinicaltrials.gov/ct2/show/NCT03086486
NCT03338621	Pulmonary TB, MDR-TB, DR-TB, DS-TB	≥18	455	BDQ, PMD, MXF, PZA	II/III	Completed	Jul 2018	Jun 2022	https://clinicaltrials.gov/ct2/show/NCT03338621
NCT03474198	Pulmonary TB	18-65	900	RFP, INH, PZA, EMB, LNZ, CFZ, RFT, LVX, BDQ	II/III	Recruiting	Mar 2018	Estimated Mar 2022	https://clinicaltrials.gov/ct2/show/NCT03474198
NCT03678688	Pulmonary TB	18-64	122	BDQ, DLM, OPC-167832	I/II	Completed	Oct 2018	Jun 2022	https://clinicaltrials.gov/ct2/show/NCT03678688
**NCT03828201**	**TB, MDR-TB**	**≥12**	**220**	**DLM, LVX, BDQ, CFZ, LNZ**	**II**	**Recruiting**	**Jun 2022**	**Estimated Jul 2025**	https://clinicaltrials.gov/ct2/show/NCT03828201
**NCT03896685**	**Pulmonary TB, MDR-TB**	**≥15**	**324**	**BDQ, DLM, LNZ, CFZ**	**III**	**Recruiting**	**Apr 2020**	**Estimated Nov 2024**	https://clinicaltrials.gov/ct2/show/NCT03896685
NCT03959566	Pulmonary TB	18-65	75	BDQ, DLM, MXF, SZD	II	Completed	May 2021	Sep 2022	https://clinicaltrials.gov/ct2/show/NCT03959566
**NCT04062201**	**Pulmonary TB, MDR-TB, XDR-TB, Pre-XDR-TB, RR-TB**	**≥6**	**402**	**BDQ, DLM, LNZ, LVX, CFZ, INH, EMB, PZA**	**III**	**Active, not recruiting**	**Aug 2019**	**Estimated Jun 2023**	https://clinicaltrials.gov/ct2/show/NCT04062201
NCT04081077	Pulmonary TB, MDR-TB, XDR-TB	≥18	240	BDQ, PMD, MXF, LNZ, CFZ	II/III	Active, not recruiting	Aug 2019	Estimated Sep 2022	https://clinicaltrials.gov/ct2/show/NCT04081077
NCT04207112	Pulmonary TB, MDR-TB, XDR-TB	≥18	200	BDQ, PMD, MXF, LNZ, CFZ	II/III	Recruiting	Oct 2020	Estimated Jul 2022	https://clinicaltrials.gov/ct2/show/NCT04207112
NCT04545788	RR-TB	18-65	200	LNZ, BDQ, CS	/	Recruiting	Aug 2020	Estimated Dec 2022	https://clinicaltrials.gov/ct2/show/NCT04545788
NCT04550832	Pulmonary TB	18-65	76	DZD, BDQ, DLM, MXF	II	Active, not recruiting	Oct 2021	Estimated Mar 2024	https://clinicaltrials.gov/ct2/show/NCT04550832
NCT04629378	TB	18-65	22	MPM, co-amoxiclav, PZA, BDQ, EMB	II	Completed	Aug 2020	Jun 2021	https://clinicaltrials.gov/ct2/show/NCT04629378
NCT05007821	Pulmonary TB, MDR-TB	≥18	132	LNZ, BDQ, DLM, CFZ	II	Recruiting	Aug 2022	Estimated Sep 2025	https://clinicaltrials.gov/ct2/show/NCT05007821
NCT05040126	Pulmonary TB, MDR-TB, Pre-XDR-TB	18-65	400	LNZ, BDQ, PMD	III	Recruiting	Oct 2021	Estimated Mar 2024	https://clinicaltrials.gov/ct2/show/NCT05040126
NCT05221502	Pulmonary TB	18-65	120	BDQ, DLM, OPC-167832	II	Recruiting	Apr 2022	Estimated Feb 2024	https://clinicaltrials.gov/ct2/show/NCT05221502
NCT05278988	MDR-TB	18-66	60	BDQ, DLM, CFZ, PZA	IV	Recruiting	Apr 2021	Estimated Sep 2024	https://clinicaltrials.gov/ct2/show/NCT05278988
NCT05306223	TB, MDR-TB	18-65	212	BDQ, LVX, LNZ, CS, CFZ, PZA, PTH	IV	Recruiting	May 2022	Estimated Aug 2025	https://clinicaltrials.gov/ct2/show/NCT05306223
NCT05382312	TB	18-65	55	GSK3036656, BDQ, DLM, EMB	II	Recruiting	Jul 2022	Estimated Sep 2023	https://clinicaltrials.gov/ct2/show/NCT05382312
NCT05556746	Pulmonary TB, HIV Infections	≥18	156	BDQ, CFZ, PZA, DLM, RFP, INH, EMB	II	Not yet recruiting	Estimated Mar 2023	Estimated Jun 2026	https://clinicaltrials.gov/ct2/show/NCT05556746
NCT05686356	TB	18-65	352	SZD, NAC, PMD, BDQ, EMB	II/III	Not yet recruiting	Estimated Jan 2023	Estimated Sep 2025	https://clinicaltrials.gov/ct2/show/NCT05686356
**NCT05766267**	**Pulmonary TB**	**≥12**	**288**	**RFB, DLM, BDQ, MXF, PZA, INH, RFP, EMB**	**II/III**	**Not yet recruiting**	**Estimated Mar 2023**	**Estimated Apr 2026**	https://clinicaltrials.gov/ct2/show/NCT05766267
NCT05807399	Pulmonary TB	18-65	360	SZD, RFP, INH, PZA, MXF, BDQ, DLM	II	Recruiting	Apr 2023	Estimated Feb 2025	https://clinicaltrials.gov/ct2/show/NCT05807399

Data until April 2023. Recent data might not have been updated. Bold black indicates clinical trials that included children. TB, tuberculosis; MDR-TB, multidrug-resistant TB; XDR-TB, extensively drug-resistant tuberculosis TB; Pre-XDR-TB, pre-extensively drug-resistant tuberculosis TB; RR-TB, rifampicin-resistant TB; BDQ, Bedaquiline (TMC-207, R207910); CFZ, Clofazimine (NSC-141046); CS, Cycloserine; DLM, Delamanid (OPC-67683); DTG, Dolutegravir; DZD, Delpazolid; EMB, Ethambutol (Rifafour e-275); ETA, Ethionamide; INH, Isoniazid; KAN, Kanamycin; LNZ, Linezolid; LVX, Levofloxacin; MPM, Meropenem; MXF, Moxifloxacin; NAC, N-acetylcysteine; PMD, Pretomanid (PA-824); PTH, Prothionamide; PZA; RFB, Rifabutin Pyrazinamide; RFP, Rifampicin(Rifampin); RFT, Rifapentine; SZD, Sutezolid; TRD, Terizidone.

A systematic review and meta-analysis conducted by Holmgaard et al. included 13 studies with a total of 1031 individuals and reported a combined estimate of a favorable treatment outcome of 73.1%. The review also found that sputum culture conversion rates at 6 months ranged from 61% to 95%. Overall, QTc prolongation was 7.8% (Holmgaard et al., 2023). Culture conversion and Treatment effectiveness of delamanid-containing regimens were evaluated by a comprehensive evaluation of 25 studies (22 observational and 3 experimental studies) including 1276 patients. In observational studies, the group of regimens including delamanid, which included 591 patients, had a treatment success rate of 80.9%; by contrast, the group of regimens combining delamanid and bedaquiline, which included 685 patients, had a success rate of 72.8%. The success rates for delamanid-containing regimens were 72.5% in experimental investigations including 411 patients (Nasiri et al., 2022). Franke et al. conducted a study that analyzed data from 1,109 patients receiving polypharmacy therapy with bedaquiline (63%), delamanid (27%), or both (10%), 939 patients (85%) experienced culture conversion within 6 months. The incidence of culture conversion was lower among HIV patients (Franke et al., 2021). However, the findings suggest that the combination of bedaquiline and delamanid is still an effective regimen for the treatment of MDR-TB, with a low incidence of clinically significant cardiotoxicity. The expanded use of this drug combination could be particularly beneficial for unique cohorts, such as TB patients with AIDS.

Unfortunately, there is a dearth of combination trials available for children, which makes it imperative to collect data on children to provide them with faster and more effective treatment options. In South Africa, an injection-free regimen containing bedaquiline and delamanid was used to treat RR-TB in adolescents (10-19 years). The final outcomes at the end of treatment for 22 participants were 17 (77%) treatment successes, 2 (9%) lost to follow-up, 2 (9%) treatment failures, and 1 (5%) death. These results indicate that injection-free regimens containing bedaquiline and/or delamanid are effective and well-tolerated in adolescents, which is consistent with WHO recommendations for this age group (Mohr-Holland et al., 2020). Another study conducted on children in Mumbai, India showed similar results; an injection-free regimen containing bedaquiline and/or delamanid was found to be effective and well tolerated when administered on an outpatient basis. Therefore, it should be routinely available to these vulnerable groups (Das et al., 2020).

While the potential QTc prolonging effects of bedaquiline and delamanid limit their combined use, there is some preliminary experience with their concomitant use in adults. In the absence of effective alternative treatments, this combination may be considered for use in children under close clinical monitoring (Matteelli et al., 2015; Seddon et al., 2012). Two children with highly DR-TB received bedaquiline and delamanid, resulting in a prolonged QTc interval when assessed using the Bazett formula. However, when assessed using the Fridericia formula, the QTc interval was normal. To ensure patients receive the maximum benefit from treatment, it is crucial to emphasize the use of appropriate monitoring formulas to assess for potential adverse effects, such as prolonged QTc intervals. If necessary, the required interventions can be promptly initiated without discontinuing treatment for patients who would otherwise benefit from it (Shah et al., 2020).

## 6 Discussion

Significant progress has been made in the development of drugs to treat MDR-TB infection in children. Bedaquiline and delamanid have been used successfully used and are recommended by WHO as part of the treatment regimen. However, further studies are required to gather accurate information on the safety and effectiveness of these drugs in pediatric patients. It is important to use anti-TB drugs judiciously until more data are available.

To accelerate the goal of TB elimination, it is critical to continue the search for effective new therapeutic targets and drugs. The development of new drugs should focus on avoiding negative drug-drug interactions, utilizing novel modes of action that reduce cross-resistance, and ensuring compatibility with other drugs that are used in combination. In addition, the use of analogs of existing drugs could provide more effective and safer treatments for TB. It is also important to explore new technologies to discover new targets and new treatments for TB, such as using CRISPR to disrupt chemical genetics platforms in order to discover new drug targets and drug resistance mechanisms (Li et al., 2022). Furthermore, the issue of phage therapy, has become increasingly serious due to concerns about antimicrobial resistance (Strathdee et al., 2023). The development of drugs to treat children with MDR-TB is a challenging task due to the lack of easy ways for children to take the adult formulations correctly, and the limited availability of medicines suitable for children. Urgent action is required to expedite research in creating child-friendly medicines that can be more accessible worldwide, thereby ensuring that children can receive appropriate treatment.

In conclusion, our review highlights the use of bedaquiline and delamanid as potential treatments for children with MDR-TB. We summarize their development history, efficacy, safety and potential adverse effects. Further research is necessary to determine the optimal use of these drugs for treating MDR-TB in children.

## Author contributions

KM conceived and designed the article; HZ and XZ wrote the manuscript; ZZ and LL put forward professional opinions; KM and JB revised the manuscript. All authors contributed to the article and approved the submitted version.
